# Genome-wide identification and expression analysis of protein arginine methyltransferase and JmjC domain-containing family in apple

**DOI:** 10.3389/fpls.2024.1381753

**Published:** 2024-05-28

**Authors:** Shenghui Su, Min Ji, Jiaqi Chen, Meidie Zhang, Xiaozhao Xu, Chenxia Cheng

**Affiliations:** ^1^ College of Horticulture, Qingdao Agricultural University, Qingdao, Shandong, China; ^2^ Engineering Laboratory of Genetic Improvement of Horticultural Crops of Shandong Province, Qingdao Agricultural University, Qingdao, China; ^3^ National Technology Centre for Whole Process Quality Control of FSEN Horticultural Products (Qingdao), Qingdao Agricultural University, Qingdao, China; ^4^ Laboratory of Quality & Safety Risk Assessment for Fruit (Qingdao), Ministry of Agriculture and Rural Affairs, Qingdao Agricultural University, Qingdao, China; ^5^ Qingdao Key Lab of Modern Agricultural Quality and Safety Engineering, Qingdao Agricultural University, Qingdao, China; ^6^ Academy of Dongying Efficient Agricultural Technology and Industry on Saline and Alkaline Land in Collaboration with Qingdao Agricultural University, Dongying, China

**Keywords:** Prmt, JMJ, apple, gene expression, gene family

## Abstract

Histone methylation is an important type of histone modification that regulates gene expression in plants. In this study, we identified 14 arginine methylation-related genes (*Protein Arginine Methyltransferase*, *MdPRMT*) and 32 demethylation-related genes (*JmjC Domain-Containing Family*, *MdJMJ*) in apple. Furthermore, we investigated the phylogenetic relationship, chromosome distribution, gene structure, motif analysis, promoter sequence analysis, and expression patterns of *MdPRMT* and *MdJMJ* genes. Homology analysis showed a high degree of conservation and homology between *PRMT* and *JMJ* genes in *Arabidopsis* and apple. We identified the types of duplicated genes in the *MdJMJ* and *MdPRMT* gene families, found a large number of whole-genome duplicates (WGD) gene pairs and a small number of tandem duplicates (TD) pairs, transposed duplication (TRD) gene pairs as well as proximal duplicates (PD) pairs, and discussed the possible evolutionary pathways of the gene families from the perspective of duplicated genes. Homology analysis showed a high degree of conservation and homology between *PRMT* and *JMJ* genes in *Arabidopsis* and apple. In addition, the promoter regions of *MdPRMT* and *MdJMJ* contain numerous *cis*-acting elements involved in plant growth and development, hormone response, and stress responses. Based on the transcriptional profiles of *MdPRMT* and *MdJMJ* in different tissues and developmental stages, it was found that *MdPRMT* and *MdJMJ* may play multiple roles in apple growth and development, for example, *MdJMJ21* may be involved in the regulation of apple endosperm formation. *MdPRMT* and *MdJMJ* exhibit different expression patterns in response to hormone signaling in apple, *MdJMJ3*, *MdJMJ18*, *MdJMJ30*, *MdPRMT2*, *MdPRMT13*, and *MdPRMT14* may play roles in apple response to drought stress, while the expression of *MdJMJ13*, *MdPRMT3*, *MdPRMT4*, and *MdPRMT6* is affected by cold stress. Our study provides a foundation for determining the functional roles of *MdPRMT* and *MdJMJ* genes in apple.

## 1 Introduction

In eukaryotes, the basic structural unit of chromatin is the nucleosome (Kouzarides, 2007). Generally, nucleosomes are composed of octamers consisting of two copies each of four histone subunits, H2B, H2A, H3, and H4 (Thomas Jenuwein, 2001; Liu et al., 2010). The N-terminal amino acid residues of histones are susceptible to post-translational modifications, including acetylation, methylation, phosphorylation, ubiquitination, and other histone modifications (Kouzarides, 2007). In recent years, with the advancement in detection techniques for histone modifications and further research, it has been discovered that the middle and C-terminal regions of histones can also be specifically modified. These modifications affect the compactness and accessibility of chromatin in different ways, thereby influencing gene expression and ultimately affecting various physiological and developmental processes in organisms. They are one of the most important epigenetic regulatory mechanisms of gene expression in eukaryotes (Lawrence et al., 2016). Histone modifications are reversible covalent modifications. The occurrence, removal, and functional roles of these covalent modifications are mainly regulated by histone-modifying enzymes and their corresponding cofactors, including Writers, Erasers, and Reader/Effectors (Liu et al., 2010). Writers are enzymes that catalyze the addition of chemical groups to histones for modification, such as histone acetyltransferases (HATs), histone methyltransferases (HMTs), kinases, and ubiquitinases. Erasers are enzymes that remove these modifications from histones, such as histone deacetylases (HDACs), histone demethylases (HDMs), phosphatases, and deubiquitinases. Readers are proteins or protein complexes that recognize and specifically bind to substrates with specific post-translational modifications (Liu et al., 2010). Among them, histone methylation, as one of the main types of histone modifications, primarily affects the binding of histones to Reader proteins, leading to changes in chromatin structure, and thus transcriptional repression or activation (Genschik et al., 2014). Histone methylation can be divided into lysine methylation and arginine methylation based on their occurrence sites (Alvarez-Venegas, 2005), so HMTs can also be classified into HKMTs and PRMTs. The removal of histone methylation modifications is mainly accomplished by HDMs (Luo et al., 2014).

Protein arginine methylation is catalyzed by type I and II PRMTs, with type I PRMTs catalyzing asymmetric dimethylation of arginine residues and type II PRMTs catalyzing symmetric dimethylation of arginine residues (Bedford and Richard, 2005). Studies have shown that *Arabidopsis* protein arginine methyltransferase 5 (AtPRMT5), a homolog of human PRMT5, is an enzyme capable of catalyzing symmetric dimethylation of arginine residues, and it plays a role in *Arabidopsis* growth and development, especially in promoting flowering (Wang et al., 2001; Pei et al., 2007). In addition, other type I PRMTs in *Arabidopsis* that can catalyze asymmetric dimethylation of arginine residues (*AtPRMT10*, *AtPRMT4a*, and *AtPRMT4b*) have also been shown to promote flowering (Niu et al., 2007, 2008). Overall, existing evidence suggests that protein arginine methyltransferases play important roles in plant growth and development. HDMs are key factors regulating the steady state of histone methylation. HDMs can be divided into two types based on their mechanisms: lysine-specific demethylase KDM1/LSD1 and demethylases containing Jumonji C (JmjC) domains. KDM1/LSD1 performs demethylation of mono- and dimethylated lysine residues through FAD-dependent amine oxidation reactions, while JmjC proteins catalyze demethylation reactions that are dependent on iron (II) and α-ketoglutarate, and they play important roles in histone demethylation. They can remove methylation modifications at H3K4, H3K9, H3K27, and H3K36 (Anand and Marmorstein, 2007). *Arabidopsis* contains 21 JmjC domain proteins (JMJs), which are divided into five subfamilies (KDM5/JARID1, KDM4/JHDM3, KDM3/JHDM2, JMJD6, and JmjC domain-only) based on protein sequence similarity (Luo et al., 2014). In rice, 20 JmjC domain proteins have been identified, among which OsJMJ706 specific to H3K9 and OsJMJ703 specific to H3K4 have been shown to be involved in the regulation of rice flower organ development and transposon silencing, respectively (Sun and Zhou, 2008; Copenhaver et al., 2013; Cui et al., 2013). These findings collectively indicate that PRMTs and HDMs play important roles in plant growth and development.

Apple (*Malus domestica*) is one of the most important fruits in the world (Perini et al., 2014). Previous studies have shown that histone lysine methylation in apples is primarily mediated by a class of proteins containing SET domains (SDGs). The apple genome contains a total of 67 *SDG* genes, which play important roles in apple development and stress responses (Li et al., 2021). However, research on protein arginine methyltransferases and histone demethylases in apples is very limited, so it is necessary to reveal their functional characteristics in apples.

In this study, a total of 32 JMJ members and 14 PRMT members were identified in the apple genome, and they were subjected to detailed analysis in terms of systematic evolution, homology relationships, conserved domains, gene structure, and *cis*-acting elements. In addition, the expression profiles of *MdPRMTs* and *MdMJMs* in different organs of apple at different developmental stages, under biotic and abiotic stresses, and in response to hormones were also studied. In conclusion, this study provides a comprehensive analysis of *JMJ* and *PRMT* genes in apple, laying a foundation for further exploration of their regulatory roles in apple development, stress responses, and hormone signaling.

## 2 Materials and methods

### 2.1 Identification and classification of histone methylation modification genes

Following previous research, the *Arabidopsis* Information Resource (TAIR, https://www.arabidopsis.org/) was used to obtain the *Arabidopsis thaliana* histone methylation modification genes protein sequences. The whole genome data of apples (*Malus domestica*) was obtained from the Apple Genome Database (http://bioinformatics.cau.edu.cn/AppleMDO/) (Da et al., 2019). And the gene identifier (ID) can be found in the **Supplementary Table S1**.

Candidate histone methylation modification genes (*PRMTs* and *JMJs*) in apple were identified using; two basic local alignment search tool (BLAST) methods implemented in TBtools (Chen et al., 2020). The *Arabidopsis* PRMTs and JMJs protein sequences were used as queries for BLAST analysis. Additionally, relevant hidden Markov models were obtained from the Pfam database (http://pfam.xfam.org/), and employed to search the apple protein sequence data using the HMMER 3.0 software with a stringent threshold of E ≤ 10–^20^. After removing redundancy and duplicate sequences, a preliminary set of candidate sequences was determined. The intersection of the gene family candidate members was obtained by combining the results obtained from the above-mentioned methods. Subsequently, the conserved protein domains of the target gene family in apples were analyzed using the Web CD-Search tool on the NCBI website (https://www.ncbi.nlm.nih.gov/Structure/bwrb/bwrpsb.cgi/). This analysis aimed to determine whether the conserved domain related to the target gene family protein was present in each candidate sequence. Only the candidate sequences containing complete domains were retained for further analysis. In the case of those sequences that contained different or incomplete domains, their sequence integrity was assessed by submitting them to the SoftBerry website (http://linux1.softberry.com/) before subjecting them to the Batch Web CD-Search tool for comparing the similarity between their domains and those of *Arabidopsis* PRMTs and JMJs sequences.

The protein physicochemical properties of gene family members were calculated using the ExPASy online software ProtParam (https://web.expasy.org/protparam/). These properties encompass various parameters such as the number of amino acids, molecular weight, theoretical pI, instability index, aliphatic index, and grand average of hydropathicity. The chromosome location of each confirmed histone methylation modification gene was retrieved from the GFF3 file of apple genome, and then visualized on apple chromosomes using TBtools (Chen et al., 2020).

### 2.2 Phylogenetic analysis of histone methylation modification proteins in *M. domestica* and *A. thaliana*

The amino acid sequences of PRMTs, and JMJs in *Arabidopsis* and apple were obtained individually from the TAIR and AppleMDO databases. These sequences were aligned using MUSCLE in MEGA 11 software. Then, the optimal model was determined using MEGA 11 software, and the phylogenetic tree was constructed using the maximum likelihood method(ML) (Tamura et al., 2021). The bootstrap consensus tree inferred from 1000 replicates is taken to represent the evolutionary history of the taxa analyzed (Felsenstein, 1985). The phylogenetic tree was visualized and optimized by TBtools.

### 2.3 Chromosomal distribution and synteny analysis

The gene location analysis was performed using the Amazing Gene Location tool in TBtools, which mapped the *PRMTs* and *JMJs* to their respective chromosomes based on the information extracted from the GTF/GFF files. To visualize the synteny blocks within the apple genome and between the genomes of apple, *Arabidopsis*, rice and maize, the Dual Synteny Plotter tool in TBtools was employed. These synteny blocks were generated using the One Step MCScanX-Super Fast method. Rice and maize genome data were both downloaded from the EnsemblPlants database (https://plants.ensembl.org/index.html). The Simple Ka/Ks calculator (NG) in TBtools was utilized to calculate the synonymous (Ks) and non-synonymous (Ka) values for duplicated genes.

### 2.4 Identification of duplicated gene types

We employed the DupGen_finder tool (https://github.com/qiao-xin/DupGen_finder) to classify the types of duplicated genes. DupGen_finder_unique categorizes plant genome duplicated genes into five categories based on a specific algorithm: whole-genome duplication (WGD), proximal duplication (PD), transposed duplication (TRD), dispersed duplication (DSD), and tandem duplication (TD) (Qiao et al., 2019). Using *Arabidopsis* as an outgroup for apple, we followed the recommended workflow of DupGen_finder for our analysis and parameter configuration as previously described (Kenchanmane Raju et al., 2023).

### 2.5 Sequence analysis

The conserved features of PRMTs and JMJs sequences were analyzed and visualized based on motifs using the Multiple Em for Motif Elicitation (MEME) suite 5.4.1 (https://meme-suite.org/meme/tools/meme) (Bailey et al., 2015) and TBtools. The gene structure of PRMT and JMJ genes were assessed using Gene Structure Shower in TBtools, utilizing information from the apple genome GFF3 file. To identify the cis-elements in the promoters of *PRMT* and *JMJ* genes, PlantCare (https://bioinformatics.psb.ugent.be/webtools/plantcare/html/) was employed (Lescot et al., 2002).

### 2.6 Expression profiling of *PRMT* and *JMJ* genes in apple

The expression pattern of *PRMT* and *JMJ* genes following abiotic, hormone and biotic stress treatment were obtained from SRA database in NCBI website or National Genomics Data Center. The study IDs were as follows: for abiotic stress treatment: salt treatment (SRP229388), cold stress (CRA002596) and drought treatment (SRP347250); for hormone treatment: 1-Methylcyclopropene (1-MCP) treatment (SRP334206), Gibberellin Acid 3 (GA_3_) and 1-Naphthaleneacetic acid (NAA) treatment (SRP185711) and Indole-3-butyric acid (IBA) treatment (SRP330812); for biotic stress treatment: *Alternaria alternata* (SRP091754), *Pythium ultimum* (SRP048684), *Fusarium solani* (SRP239526) and ring rot (SRP153065). Kallisto, an RNA-seq quantification program were used to calculate the expression of gene (Bray et al., 2016). In this study, the gene expression was estimated using the fragment number per kilobase per million mapped exons (FPKM) and transcripts per kilobase of exon model per million mapped reads (TPM) method.

### 2.7 Quantitative reverse transcription polymerase chain reaction

Apple (‘GL3’) tissue culture seedlings were transferred to MS medium plus 200 mM sodium chloride (NaCl), 10% polyethylene glycol (PEG) 6000. Leaves were sampled at 0, 6, 12 and 24h after treatment for RNA isolation. Total RNA was extracted from apple leaves using the hot borate method as previously described (Ma et al., 2006). For reverse transcription-PCR, first-strand cDNA was synthesized from 1 μg of total RNA using Reverse Transcriptase M-MLV (RNase H-) (Takara Biomedical Technology Co., Ltd., Shiga, Japan). qRT-PCR was conducted with Takara SYBR Premix Ex Taq II (Takara Biomedical Technology Co., Ltd.) using a Light Cycler 480 instrument (Roche, Basel, Switzerland). *MdEF-1α* were used as internal controls. All primers used are listed in **Supplementary Table S8**.

## 3 Results

### 3.1 Identification of *PRMTs* and *JMJs* in apple

To identify the *PRMTs* and *JMJs* gene families in the apple genome, a comprehensive investigation was carried out using the protein sequences of the corresponding genes from *Arabidopsis* as search queries. After eliminating redundant and duplicate sequences, we identified 14 and 32 genes in PRMT and JMJ families, respectively, in the apple genome (**Table 1**). The predicted protein sequence lengths of the PRMT and JMJ families ranged from 232 (MdPRMT11) to 722 (MdPRMT3) and 187 (MdJMJ2) to 1843 (MdJMJ14) amino acids, respectively. The molecular weight (MW) of the PRMT and KDM families ranged from 25.45 (MdPRMT11) to 81.12 (MdPRMT3) and 21.43 (MdJMJ2) to 210 (MdJMJ14) kDa, respectively. The isoelectric point (pI) values for the PRMT and JMJ families varied from 25.45 (MdPRMT11) to 81.12 (MdPRMT3) and 5.05 (MdJMJ8) to 9.03 (MdJMJ28), respectively. The complete information about the genes, including gene locus ID, chromosomal position, coding sequence length, and protein sequence length, was shown in **Table 1**. The nomenclature of the genes followed a sequential order based on their arrangement on the chromosome.

**Table 1:** *PRMT and JMJ* genes identified in apple.

Gene family	Gene name	Gene ID	Chro mosome	Genomiclocation	GRAVY	Strand	Length(aa)	pI	MW(kDa)
PRMTs	*MdPRMT1*	MD02G1037100	chr2	2,800,117–2,803,571	-0.455	+	383	5.43	43.09
	*MdPRMT2*	MD02G1074300	chr2	5,963,796–5,966,843	-0.261	+	393	5.11	44.16
	*MdPRMT3*	MD04G1063800	chr4	8,432,676–8,437,714	-0.216	+	722	5.99	81.12
	*MdPRMT4*	MD07G1046100	chr7	3,921,653–3,932,752	-0.325	–	636	4.6	70.02
	*MdPRMT5*	MD08G1132700	chr8	12,539,643–12,547,230	-0.259	+	649	5.55	72.7
	*MdPRMT6*	MD13G1002200	chr13	136,755–142,344	-0.4	–	423	5.9	47.63
	*MdPRMT7*	MD13G1168500	chr13	13,630,857–13,636,161	-0.284	–	545	5.31	60.72
	*MdPRMT8*	MD15G1111800	chr15	7,855,147–7,857,854	-0.098	–	241	5.33	27.34
	*MdPRMT9*	MD15G1112100	chr15	7,859,494–7,866,979	-0.244	–	649	5.62	72.56
	*MdPRMT10*	MD15G1112600	chr15	7,919,669–7,923,893	-0.189	–	421	5.87	47.34
	*MdPRMT11*	MD15G1112700	chr15	7,923,895–7,927,030	-0.077	–	232	4.61	25.45
	*MdPRMT12*	MD15G1177300	chr15	13,877,192–13,880,754	-0.381	+	382	5.28	42.85
	*MdPRMT13*	MD15G1203800	chr15	16,210,542–16,213,676	-0.266	+	391	5.06	43.91
	*MdPRMT14*	MD16G1168900	chr16	14,047,574–14,053,079	-0.228	–	552	5.33	61.36
JMJs	*MdJMJ1*	MD00G1097500	chr0	20,399,985–20,405,925	-0.548	–	1042	5.63	117.30
	*MdJMJ2*	MD00G1097600	chr0	20,412,328–20,413,454	-0.473	–	187	5.73	21.43
	*MdJMJ3*	MD01G1082300	chr1	18,879,413–18,889,690	-0.921	–	1099	8.51	126.12
	*MdJMJ4*	MD01G1103000	chr1	21,501,838–21,506,502	-0.213	–	903	8.97	97.96
	*MdJMJ5*	MD01G1106000	chr1	21,918,410–21,925,954	-0.55	–	1603	5.67	176.95
	*MdJMJ6*	MD01G1218500	chr1	31,068,432–31,075,270	-0.681	+	1519	8.34	166.66
	*MdJMJ7*	MD03G1220300	chr3	30,449,337–30,452,592	-0.195	–	748	6.6	82.31
	*MdJMJ8*	MD04G1202800	chr4	28,862,118–28,866,548	-0.457	+	600	5.05	67.77
	*MdJMJ9*	MD04G1229800	chr4	30,997,445–31,004,908	-0.591	–	1215	6.36	135.97
	*MdJMJ10*	MD05G1326700	chr5	45,286,027–45,292,287	-0.562	–	1024	8.43	116.05
	*MdJMJ11*	MD05G1351300	chr5	46,832,063–46,837,369	-0.677	–	887	6.07	101.73
	*MdJMJ12*	MD06G1012500	chr6	1,590,799–1,599,810	-0.869	–	1046	7.31	118.43
	*MdJMJ13*	MD06G1026100	chr6	3,210,489–3,217,400	-0.509	+	890	7.09	99.49
	*MdJMJ14*	MD06G1081900	chr6	20,068,477–20,088,135	-0.316	–	1843	6.52	210.00
	*MdJMJ15*	MD06G1159300	chr6	30,080,831–30,088,073	-0.719	–	1467	8.87	164.00
	*MdJMJ16*	MD07G1099600	chr7	10,973,691–10,988,237	-0.829	–	1233	5.98	139.42
	*MdJMJ17*	MD07G1172400	chr7	24,961,209–24,968,468	-0.535	–	1594	5.84	176.35
	*MdJMJ18*	MD08G1004400	chr8	412,295–414,742	-0.204	+	815	5.28	89.09
	*MdJMJ19*	MD08G1186800	chr8	23,531,368–23,540,505	-0.317	+	981	5.32	111.64
	*MdJMJ20*	MD10G1182700	chr10	27,556,246–27,561,506	-0.568	+	885	7.09	98.57
	*MdJMJ21*	MD10G1241100	chr10	33,658,731–33,664,694	-0.537	–	1040	5.53	117.64
	*MdJMJ22*	MD10G1304800	chr10	39,112,395–39,118,408	-0.604	–	1030	8.57	116.82
	*MdJMJ23*	MD10G1325700	chr10	40,607,236–40,611,894	-0.578	–	689	5.8	79.21
	*MdJMJ24*	MD12G1046300	chr12	5,202,290–5,205,217	-0.357	–	516	6.78	58.43
	*MdJMJ25*	MD12G1216600	chr12	29,418,485–29,426,130	-0.636	+	946	5.75	107.82
	*MdJMJ26*	MD12G1246900	chr12	31,721,067–31,728,936	-0.593	–	1236	7.14	138.65
	*MdJMJ27*	MD14G1103700	chr14	15,999,174–16,014,561	-0.304	–	1842	7.67	209.35
	*MdJMJ28*	MD14G1165600	chr14	25,922,669–25,930,130	-0.693	–	1466	9.03	163.77
	*MdJMJ29*	MD14G1175900	chr14	26,919,946–26,923,646	-0.416	+	474	5.32	55.23
	*MdJMJ30*	MD15G1003800	chr15	233,872–236,319	-0.209	+	815	5.14	88.85
	*MdJMJ31*	MD15G1372700	chr15	45,518,685–45,527,976	-0.287	+	975	5.29	110.42
	*MdJMJ32*	MD16G1280000	chr16	38,164,869–38,171,753	-0.454	–	888	6.91	99.22

### 3.2 Analysis of chromosomal distribution and evolutionary relationships of *MdJMJ* and *MdPRMT* genes

**Figure 1:**
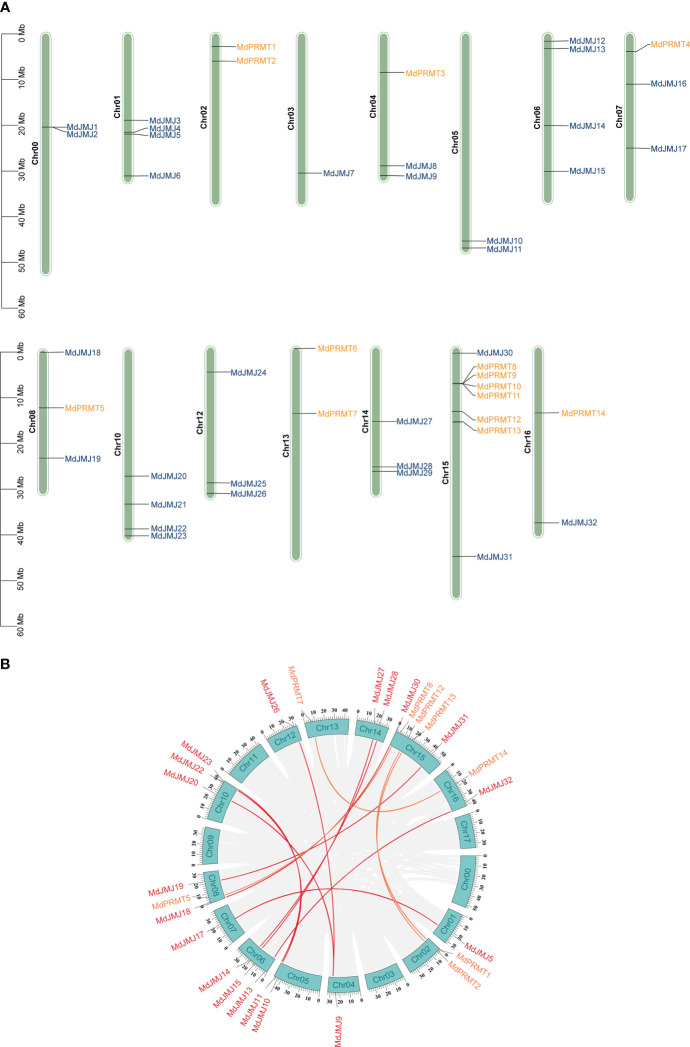
Chromosomal distribution and collinearity analysis of *JMJ* and *PRMT* genes between apple and *A. thaliana*. **(A)** Chromosomal distribution and duplication analysis of *JMJs* and *PRMTs* in apple. **(B)** Collinearity analysis of *JMJ* and *PRMT* genes family in apple.

In **Figure 1A**, the chromosomal distribution of *PRMTs*, and *JMJs* in apple was shown. The analysis revealed that the distribution of these genes is irregular. Particularly, the *MdJMJ* genes were distributed on all chromosomes apart from chromosomes 2, 9, 11, and 17. The chromosomal distribution of *MdPRMT* genes was comparatively more restricted, limited to chromosomes 2, 4, 7, 8, 13, 15, and 16. Notably, chromosome 15 showed the most concentrated distribution with a total of 6 genes (*MdPRMT8*, *MdPRMT9*, *MdPRMT10*, *MdPRMT11*, *MdPRMT12* and *MdPRMT13*).

Gene duplication events are a common occurrence across all species, playing a crucial role in generating novel functional genes and driving species evolution (Xu et al., 2012). In order to explore gene duplication events within the *JMJ* and *PRMT* gene families in apple, we conducted genome synteny analysis. The analysis revealed the presence of 10 potential duplicated genes in the *MdJMJ* gene family and 4 in the *MdPRMT* gene family (**Figure 1B**). This finding suggests that the expansion of the *MdJMJ* gene family is primarily driven by segmental duplication, as indicated by the high number of potential duplicated genes. Notably, there is a significantly gene cluster located on chromosome 15 for the *MdPRMT* gene family (**Figure 1A**).

Using *Arabidopsis* as an outgroup for apple, we followed the recommended workflow of DupGen_finder and found that after the divergence of the two species, the *MdJMJ* family contained 10 WGD-pairs, 1 TD-pairs (*MdJMJ1*-*MdJMJ2*), and 3 TRD-pairs (*MdJMJ3*-*MdJMJ6*, *MdJMJ4*-*MdJMJ7*, *MdJMJ11-MdJMJ12*). No DSD-pairs and PD-pairs were found in the *MdJMJ* family. The *MdPRMT* family had 4 WGD-pairs (*MdPRMT7- MdPRMT14*, *MdPRMT5-MdPRMT8*, *MdPRMT2*-*MdPRMT13*, *MdPRMT1-MdPRMT12*), 2 PD-pairs (*MdPRMT11*-*MdPRMT9*, *MdPRMT9*-*MdPRMT10*) and 1 TRD- pairs (*MdPRMT6*-*MdPRMT13*). No DSD-pairs and TD-pairs were found in the *MdPRMT* family. Detailed analytical results can be found in **Supplementary Table S9**. The analysis outcomes indicate that the primary mode of evolution for the *MdJMJ* and *MdPRMT* gene families is whole-genome duplication.

**Figure 2:**
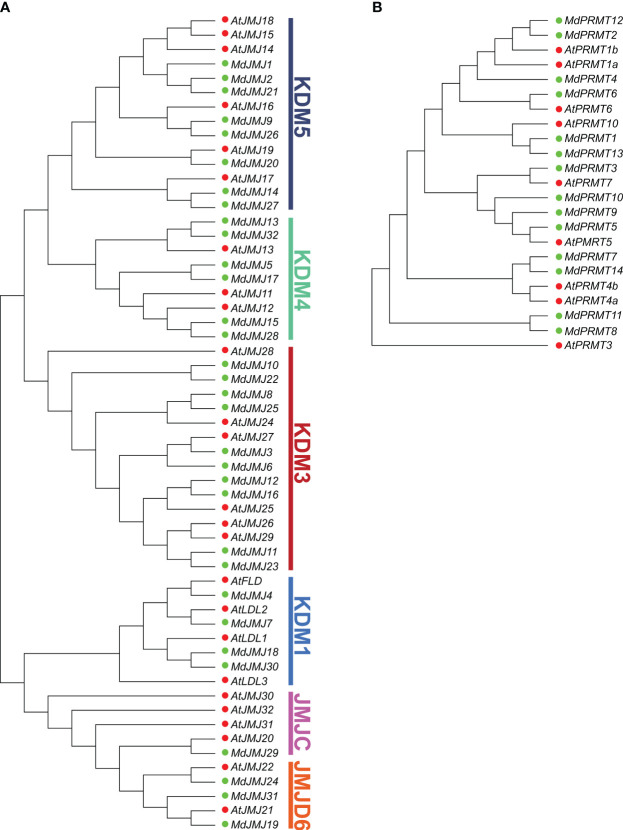
Phylogenetic relationship of JMJ **(A)** and PRMT **(B)** proteins from apple and *Arabidopsis*. The bootstrap consensus of tree was inferred from 1000 replicates, and the phylogenetic tree was constructed using the maximum likelihood method. In the figure, red dots represent *Arabidopsis* proteins, while green dots represent apple proteins.

To elucidate the evolutionary relationships of the *PRMT* and *JMJ* gene families, a maximum likelihood (ML) phylogenetic tree was constructed using the PRMT and JMJ proteins from apple and *Arabidopsis* (**Figure 2**). The classification of the gene families was based on the established *Arabidopsis* JMJ gene families. The JMJ subfamily includes KDM5, KDM4, KDM3, KDM1, JMJC, and JMJD6, with 8, 6, 10, 4, 1, and 3 MdJMJ proteins, respectively (**Figure 2A**).

**Figure 3:**
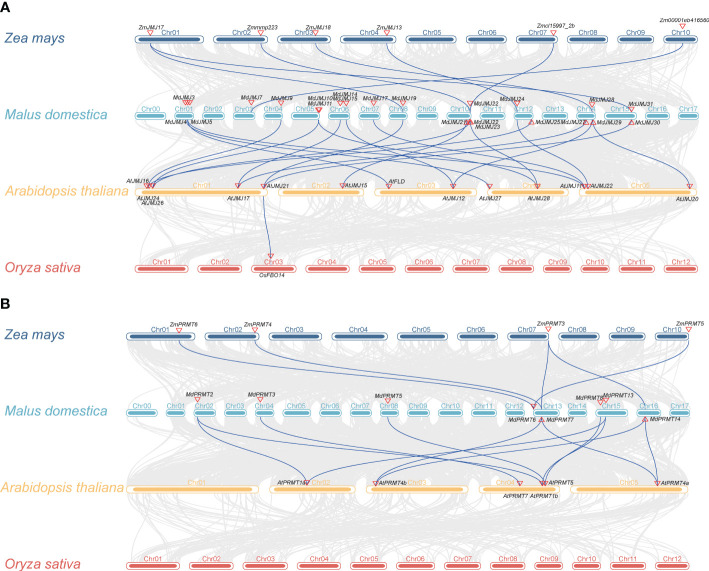
Synteny analysis of *JMJs* **(A)** and *PRMTs* **(B)** of apple, *Arabidopsis*, maize and rice.

To gain deeper insights into the origin, evolutionary history, and functional characteristics of *PRMT* and *JMJ* genes, we performed a comparative synteny analysis between the genomes of apple, *Arabidopsis*, rice and maize (**Figure 3**). The apple genome contained 19 *JMJ* genes with high synteny to the *Arabidopsis* genome, forming a total of 21 syntenic gene pairs, as shown in **Figure 3A**. These syntenic gene pairs include *MdJMJ10*-*AtJMJ16*, *MdJMJ11*-*AtJMJ17*, *MdJMJ11*-*AtJMJ18*, *MdJMJ14*-*AtJMJ19*, *MdJMJ15*-*AtJMJ20*, *MdJMJ17*-*AtJMJ21*, *MdJMJ19*-*AtJMJ22*, *MdJMJ21*-*AtJMJ23*, *MdJMJ22*-*AtJMJ24*, *MdJMJ23*-*AtJMJ25*, *MdJMJ23*-*AtJMJ26*, *MdJMJ24*-*AtJMJ27*, *MdJMJ25*-*AtJMJ28*, *MdJMJ27*-*AtJMJ29*, *MdJMJ28*-*AtJMJ30*, *MdJMJ29*-*AtJMJ31*, *MdJMJ3*-*AtJMJ32*, *MdJMJ31*-*AtJMJ33*, *MdJMJ4*-*AtJMJ34*, *MdJMJ5*-*AtJMJ35*, and *MdJMJ9*-*AtJMJ36* (**Figure 3A**). 7 syntenic gene pairs were found between apples and maize, only one ortholog pair (*AtJMJ21*-*OsFBO14*) were found in *Arabidopsis* and rice. In addition, there were 7 *PRMT* genes in the apple genome that showed synteny with the *Arabidopsis* genome, forming a total of 11 syntenic gene pairs. These syntenic gene pairs include *MdPRMT13*-*AtPRMT1a*, *MdPRMT13*-*AtPRMT1b*, *MdPRMT14*-*AtPRMT4b*, *MdPRMT14*-*AtPRMT4a*, *MdPRMT2*-*AtPRMT1a*, *MdPRMT2*-*AtPRMT1b*, *MdPRMT3*-*AtPRMT7*, *MdPRMT5*-*AtPRMT5*, *MdPRMT7*-*AtPRMT4b*, *MdPRMT7*-*AtPRMT4a*, and *MdPRMT8*-*AtPRMT5* (**Figure 3B**). Five pairs of co-linear genes (*ZmPRMT6*-*MdPRMT6*, *ZmPRMT3*-*MdPRMT7*, *ZmPRMT3*-*MdPRMT14*, *ZmPRMT4*-*MdPRMT7*, and *ZmPRMT5*-*MdPRMT6*) were identified between maize and apple, while no co-linear genes were found between *Arabidopsis* and rice. These results indicate a high conservation of *JMJ* and *PRMT* genes between the apple and *Arabidopsis* genomes.

For evolutionary studies, Ka (non-synonymous substitution) and Ks (synonymous substitution) values can be used to predict the selective pressure on duplicated genes (Zhao et al., 2016). When the Ka/Ks ratio falls below 1, it signifies that the genes have undergone purifying selection, indicating the absence of significant functional differentiation among these genes during the evolutionary process (Hurst, 2002). We utilized the Simple Ka/Ks CalculatorNG program in TBtools to analyze 14 pairs of collinear genes in *MdJMJ* and *MdPRMT* to further investigate the evolutionary relationships among these collinear genes. The results showed that all the collinear gene pairs had a Ka/Ks ratio less than 1, indicating that these genes have undergone purifying selection and have not experienced functional differentiation (**Table 2**). However, it is important to note that a Ka/Ks ratio less than 1 does not imply that these collinear genes have not undergone functional differentiation or evolution. It simply suggests that, within the observed time scale, the functional differences among these genes have had a relatively minor impact on the evolutionary process.

**Table 2:** Calculation of synonymous (Ks) and non-synonymous (Ka) for duplicated genes in apple.

Gene 1	Gene 2	Ka	Ks	Ka/Ks
*MdJMJ5*	*MdJMJ17*	0.06	0.17	0.35
*MdJMJ9*	*MdJMJ20*	0.44	1.84	0.24
*MdJMJ9*	*MdJMJ26*	0.05	0.16	0.29
*MdJMJ10*	*MdJMJ22*	0.04	0.15	0.28
*MdJMJ11*	*MdJMJ23*	0.05	0.16	0.29
*MdJMJ15*	*MdJMJ28*	0.04	0.14	0.29
*MdJMJ14*	*MdJMJ27*	0.05	0.15	0.32
*MdJMJ13*	*MdJMJ32*	0.06	0.14	0.42
*MdJMJ19*	*MdJMJ31*	0.04	0.20	0.22
*MdJMJ18*	*MdJMJ30*	0.03	0.15	0.18
*MdPRMT2*	*MdPRMT13*	0.02	0.15	0.12
*MdPRMT1*	*MdPRMT12*	0.02	0.18	0.10
*MdPRMT5*	*MdPRMT8*	0.10	0.26	0.37
*MdPRMT7*	*MdPRMT14*	0.02	0.16	0.14

### 3.3 Sequence and structure analysis of JMJs and PRMTs

**Figure 4:**
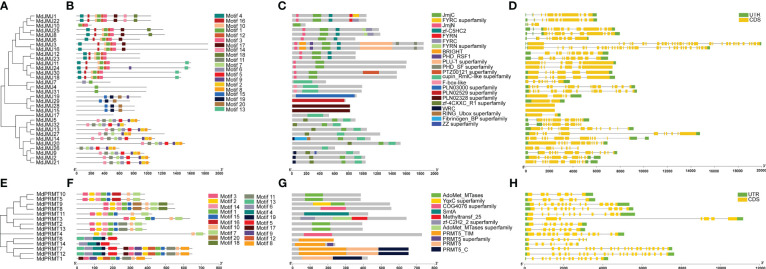
Phylogenetic analysis, motif composition, conserved domains of MdJMJ, and MdPRMT proteins and exon-intron structures of *MdJMJ* and *MdPRMT* genes. **(A–C)** The phylogenetic tree **(A)**, motif composition **(B)** and the distribution of conserved domains **(C)** of MdJMJs. **(D)** Gene structures of the *MdJMJ* genes. **(E–G)** The phylogenetic tree **(E)**, motif composition **(F)** and the distribution of conserved domains **(G)** of MdPRMTs. **(H)** Gene structures of the *MdPRMT* genes. Green rectangles represent untranslated regions (UTRs); yellow rectangles represent coding sequence (CDS) or exons; grey lines represent introns.

A phylogenetic tree for the MdJMJ and MdPRMT gene families was constructed using the Maximum Likelihood method in MEGA11.0 software (**Figures 4A, E**). It is evident that the genes clustered together in both gene families exhibit similar conserved domains. MdJMJ4 and MdJMJ31 contained only motif 1, while MdJMJ17 lacked any motifs. Additionally, motif 15, motif 19, and motif 20 were unique to MdJMJ19, MdJMJ29, MdJMJ28, and MdJMJ15, respectively (**Figures 4B**). The motif analysis was further supported by conserved domain analysis further supported the similar domain structures within gene cluster, which showed MdJMJ19, MdJMJ29, MdJMJ28, and MdJMJ15 possessed the ZZ superfamily domain, PLN02529 superfamily domain, and PLN02328 superfamily domain, respectively (**Figures 4C**). These three conserved domains were specific to these four genes, consistent with the motif analysis. Gene structure analysis revealed significant variation in the structure of *MdJMJ* genes, with clustering genes showing similar structures (**Figure 4D**). The number of introns ranged from 0 to 32, with *MdJMJ28* and *MdJMJ15* lacking introns, while *MdJMJ3* and *MdJMJ16* had the highest number of 32 introns.

For the MdPRMT gene family, which had a smaller number of members, clustering of genes based on conserved motifs revealed similar motif structures within the clusters (**Figures 4F**). Conserved domain analysis clearly reveals that MdPRMT10, MdPRMT5, MdPRMT2, and MdPRMT13 genes only contain the AdoMet_MTases conserved domain (**Figures 4G**). MdPRMT4 and MdPRMT8 genes only possess the COG4076 superfamily conserved domain. The PRMT5_C and PRMT5 conserved domains are present only in MdPRMT7, MdPRMT12, and MdPRMT1 genes. All the members of the *MdPRMT* gene family have gene structures with introns, ranging from 6 (*MdPRMT10* and *MdPRMT5*) to 22 (*MdPRMT7* and *MdPRMT12*), indicating variations in gene structure within this gene family (**Figures 4H**).

The specific amino acid sequences of motifs of MdJMJ, MdPRMT and the detailed information of the MEME sites analysis were shown in the **Supplementary Tables S2** and **S3**, respectively.

### 3.4 *Cis*-elements in the promoter of *MdPRMTs* and *MdJMJs*

**Figure 5:**
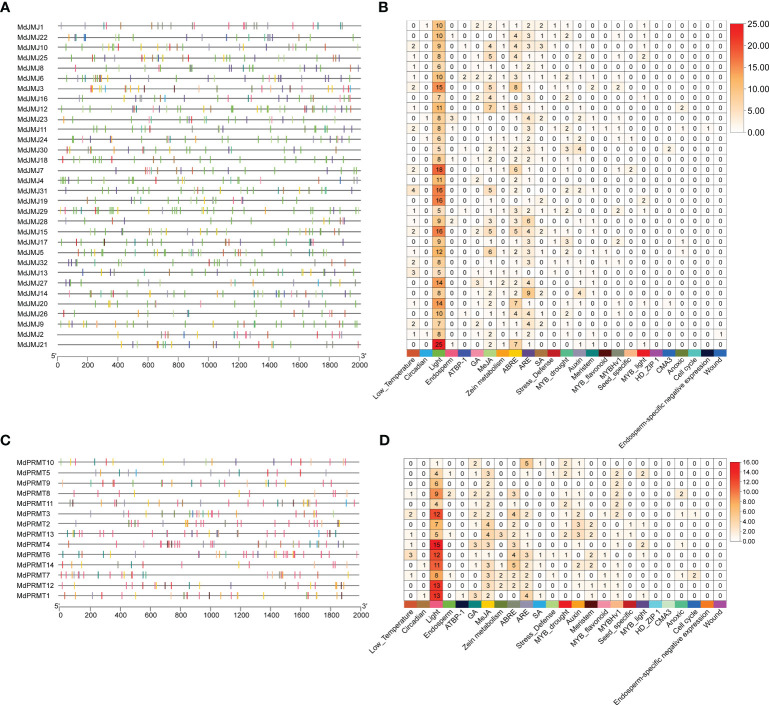
The *cis*-elements analysis of *MdJMJ* and *MdPRMT* promoters. **(A, B)** The distribution **(A)** and number **(B)** of *cis*-elements in the promoter of each *MdJMJ* genes. **(C, D)** The distribution **(C)** and number **(D)** of cis-elements in the promoter of each *MdPRMT* genes.

An analysis was conducted on the promoter fragments (-2000 bp) of all *MdPRMT* and *MdJMJ* genes (**Figures 5A, C**). A large number of *cis*-regulatory elements were identified in their promoter regions, mainly including light response, cell cycle, low temperature, circadian rhythm, stress defense, growth and development (endosperm, seed-specific, meristem, cell differentiation), hormone signaling (MeJA, ABA, GA, Auxin, and SA), anoxic specific inducibility, and some transcription factors (TF), including MYB and ATBP. Additionally, we also found a wound responsive element in each of the *MdJMJ*. By counting the number of *cis*-regulatory elements (**Figures 5B, D**), it was found that light-responsive *cis*-elements were present in the promoter regions of all members of the *MdPRMT* and *MdJMJ*. MeJA, ABRE, and ARE elements were also widely distributed. Therefore, it can be inferred that *MdPRMTs* and *MdJMJs* play important roles in apple growth and development as well as hormone responses. On the other hand, less abundant *cis*-regulatory elements should not be overlooked. For example, the low-temperature elements were present in a significant number of *MdJMJs* and *MdPRMTs*, with 18 and 6 genes involved, respectively. Stress defense elements were found in 11, and 3 genes, while MYB-drought elements were present in 20 and 8 genes of *MdJMJ* and *MdPRMT*, respectively. These genes may play crucial roles in stress tolerance in apple. Additionally, one gene in *MdJMJs* contained an Endosperm-specific negative expression element and a Wound responsive element, while none of the *MdPRMTs* genes contained these two elements.

Similarly, to compare the differences in *cis*-regulatory elements between apple and *Arabidopsis*, the same method was used to construct a map of *cis*-regulatory elements in *Arabidopsis* promoters and categorize them according to the phylogenetic tree (**Supplementary Figure S2**). The results indicate that, on average, *Arabidopsis* has a fewer variety of *cis*-regulatory elements compared to apple. Like apple, *Arabidopsis AtJMJ* and *AtPRMT* genes also contain a large number of light-responsive and stress-response elements, which can provide some assistance for the function and evolution of the apple gene families.

### 3.5 Expression profiles of the apple *MdPRMT* and *MdJMJ* in various organs, tissues and developmental stage

**Figure 6:**
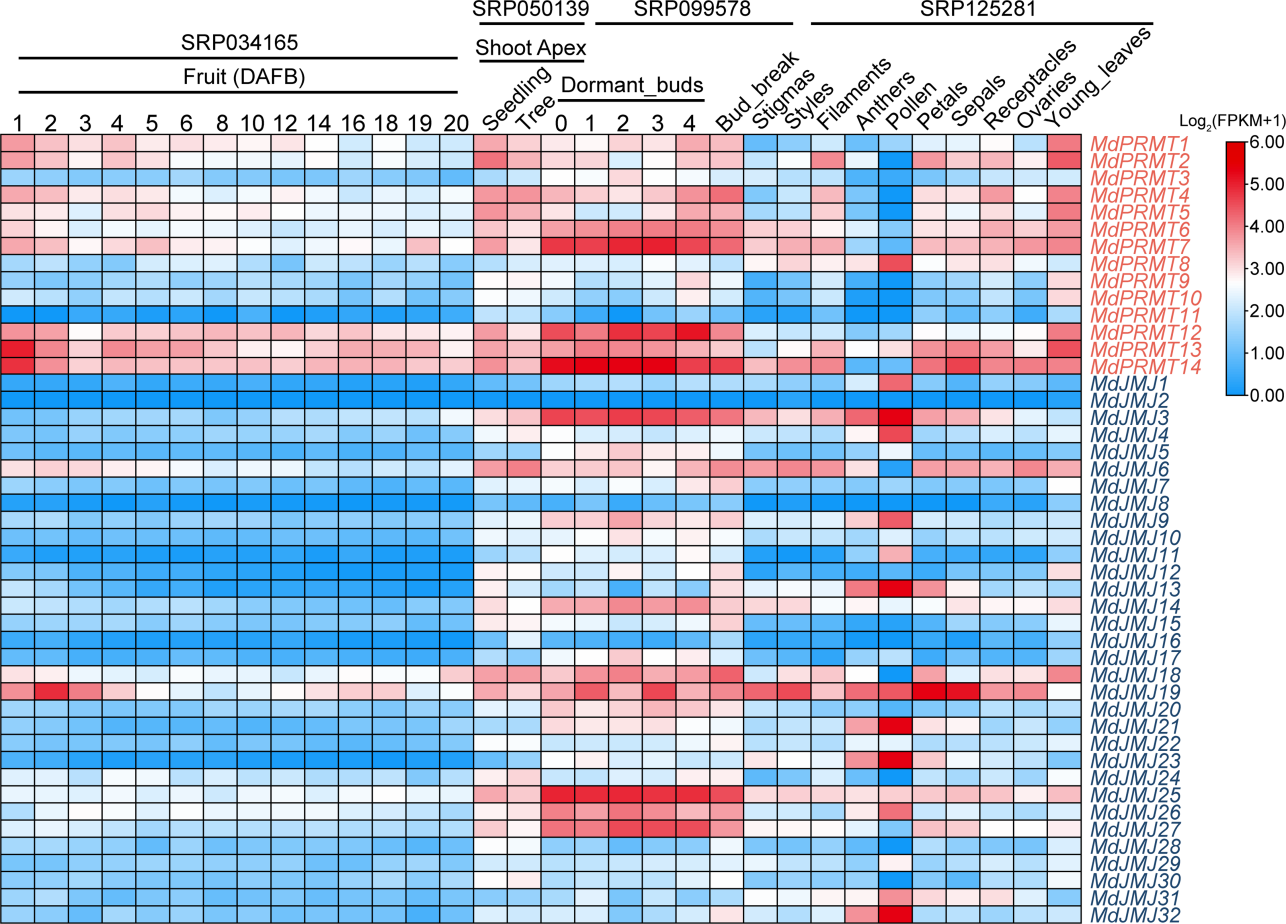
Expression profiles of the apple *MdPRMT* and *MdJMJ* genes in various organs, tissues and developmental stages. Different shades of red and blue denote the extent of the expression values according to the color bar provided [log_2_(FPKM+1)].

In this study, the expression profiles of *MdJMJs* and *MdPRMTs* in different tissues and developmental stages of apple were analyzed using publicly available transcriptome data (**Figure 6**). According to the results, the majority of histone modification-related genes showed relatively low expression levels during fruit maturation (SRP034165). It is worth noting that 11 histone modification-related genes (*MdPRMT1, MdPRMT2, MdPRMT4*, *MdPRMT5*, *MdPRMT6*, *MdPRMT7*, *MdPRMT12*, *MdPRMT13*, *MdPRMT14*, *MdJMJ6*, *and MdJMJ19*) exhibited higher expression levels in the early stage of fruit maturation, and their expression levels decreased to varying degrees as the fruit matured. Among them, the *MdPRMT* gene family had the largest number of genes, with approximately 64% of the genes showing decreased expression levels during fruit maturation. To delineate the expression patterns of histone modification-related genes in major organs of apple, transcriptome data from seedling and tree shoot apex, dormant buds and bud break, and different parts of apple flowers were analyzed. In shoot apex tissues at different stages, members of each gene family exhibited different expression patterns (SRP050139). For example, most members of the *MdPRMT* gene family showed varying degrees of down-regulation during the transition from the seedling stage to the mature stage, which was consistent with the down-regulated genes during fruit maturation. This suggests that the *MdPRMTs* may play an important role in the growth and development of apple. Furthermore, during apple plant development, 6 down-regulated genes were identified in the *MdJMJs*. Thus, it can be inferred that they collectively play a role in apple plant development.

Dormant buds play a crucial role in the growth cycle of plants and serve as a survival strategy for apple trees under adverse conditions such as winter or drought (Rohde and Bhalerao, 2007). They are also an important means of reproduction for apple trees. We analyzed the expression patterns of histone modification-related genes in apple dormant buds during dormancy and bud break (SPR099578). The results showed that *MdPRMTs* and *MdJMJs* had 5 and 6 genes, respectively, with higher expression levels. Among them, *MdPRMT7*, *MdJMJ3* and *MdJMJ25* showed relatively minor down-regulation, while the majority of genes exhibited lower expression levels or irregular trends.

Flowers are important reproductive organs of apple. We analyzed the transcriptome data from different parts of apple flowers. The results showed that histone modification-related genes had significantly higher average expression levels in pollen compared to other parts. *MdPRMTs*, and *MdJMJs* had 1 (*MdPRMT8*) and 5 (*MdJMJ3*, *MdJMJ13*, *MdJMJ21*, *MdJMJ23*, *MdJMJ32*) genes with significantly higher expression levels in pollen, respectively. Furthermore, *MdJMJ19* was expressed in all parts of the flower, with significantly higher expression levels in petals and sepals compared to other parts. Finally, it is noteworthy that *MdJMJ2* showed either no expression or relatively low expression levels in fruit development, shoot apex, bud, different parts of flowers, and young leaves.

### 3.6 Expression profiles of apple *MdPRMTs* and *MdJMJs* under different biotic and abiotic stresses

In East Asia, *Alternaria alternata* apple pathotype (AAAP) is one of the main pathogens causing apple *Alternaria* blotch disease, significantly affecting the growth and development of apple trees and fruit yield (Abe et al., 2010). Some *MdPRMTs* and *MdJMJs* showed decreased expression levels after infection, such as *MdJMJ6*, *MdJMJ15*, and *MdJMJ28*. Some genes showed an upregulation, such as *MdPRMT1*, *MdPRMT12* and *MdJMJ25*. In addition, certain genes demonstrated fluctuating expression levels, such as *MdPRMT14* and *MdPRMT7*, which increased in expression level from 0 to 18 hours after infection and then decreased below the expression level at 0 hours of infection. On the other hand, *MdJMJ27* and *MdJMJ3* showed a decrease in expression level at the early stage of infection and then an increase in expression level after 18 hours of infection. It is worth noting that the expression level of *MdJMJ13* was significantly higher at 18 and 72 hours after infection compared to other time points.

*Pythium ultimum* is one of the main pathogens causing Apple Replant Disease (ARD), which leads to growth inhibition and even death of apple seedlings when planted in orchard soil previously used for apple (or closely related species) cultivation (Zhu and Saltzgiver, 2020). The expression levels of *MdJMJ* and *MdPRMT* genes generally do not show significant changes after infection. However, it should be noted that *MdJMJ13* expression is higher than the control after 4 hours of infection, while *MdJMJ18* expression is lower than the control.

ARD causes severe growth and developmental obstacles in apple trees, and previous studies have determined *Fusarium solani* as the main pathogen causing ARD (Liu et al., 2022). There was a significant downregulation of *MdJMJ3*, *MdJMJ18*, *MdJMJ25*, *MdJMJ27*, *MdJMJ29* and *MdPRMT8*, while *MdPRMT2* showed an upregulation after *F.solani* infection. These results suggest that the above genes may play a role in apple resistance against *F. solani* infection.

**Figure 7:**
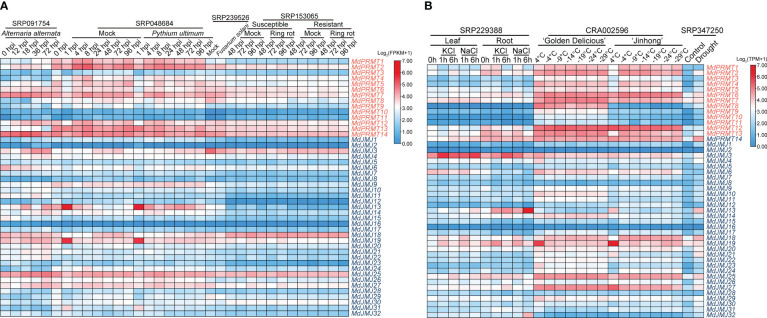
Expression profiles of the apple *MdPRMT* and *MdJMJ* genes under stress treatment. **(A)** Expression profiles of *MdPRMT* and *MdJMJ* genes at different time points under biotic stress (*Alternaria alternata*, *Pythium ultimum*, *Fusarium solani*, Ring rot) (SRP091754, SRP048684, SRP239526, SRP153065). **(B)** Expression profiles of *MdPRMT* and *MdJMJ* genes in leaves and roots at different time points under KCl and NaCl salt treatment (SRP229388), RNA-seq transcriptomes of barks (epidermis, phloem, and cambium) from one-year-old branches of two apple cultivars (‘Golden Delicious’ and ‘Jinhong’) under chilling and freezing treatments (CRA002596), and ‘GL3’ under drought stress (SRP347250). Different shades of red and blue denote the extent of the expression values according to the color bar provided [log_2_(FPKM+1) and log_2_(TPM+1)].

Apple fruit ring rot (FRR) is a highly destructive disease caused by *Botryosphaeria dothidea*, which severely affects the apple industry’s development in the Asian region (Shen et al., 2019). The results revealed that, regardless of susceptible or resistant varieties, the expression levels of *MdPRMTs* and *MdJMJs* showed no significant changes compared to the corresponding mock after *Botryosphaeria dothidea* infection (**Figure 7A**).

First, in terms of abiotic stress, we focused on salt stress (SRP229388) (**Figure 6B**). Transcriptional data from apple seedlings subjected to two types of salt stress, KCl and NaCl, showed that the expression levels of most *MdPRMTs* and *MdJMJs* were relatively low in the leaves. It is worth noting that *MdJMJ6* showed a downregulation trend in the leaves under both types of salt stress. Conversely, *MdJMJ3*, *MdJMJ13*, *MdJMJ19*, and *MdPRMT7* exhibited significant upregulation under both KCl and NaCl salt stress, with *MdJMJ3* showing higher expression levels during the initial 1 hour of KCl salt stress, followed by a significant decrease after 6 hours, while the opposite trend was observed under NaCl salt stress. In the root system, *MdJMJ13* displayed an obvious upregulation trend under both KCl and NaCl salt stress, with a significant increase in expression levels after 6 hours of NaCl stress, much higher than the expression levels under the same period of KCl stress. *MdPRMT12* showed a downregulation trend, and *MdJMJ19* exhibited an initial upregulation followed by a decrease in expression levels. A comparison between the leaf and root systems also revealed that the expression levels of *MdJMJ13*, *MdPRMT2*, *MdPRMT13*, and *MdPRMT14* under both KCl and NaCl salt stress were significantly higher in the root system than in the leaf system, whereas the opposite was observed in *MdJMJ6* and *MdPRMT7*. Neither *MdJMJ16* nor *MdJMJ2* showed expression.

In the northern region of China, low-temperature freeze injury is one of the common diseases in apple during winter and spring (Yang et al., 2011). We analyzed the RNA-seq transcriptomes of barks (epidermis, phloem, and cambium) from one-year-old branches of two apple cultivars, ‘Golden Delicious’ (non-cold-tolerant varieties) and ‘Jinhong’ (cold-tolerant varieties) under chilling and freezing treatments (CRA002596) (**Figure 7B**). The results showed that the expression level of *MdPRMT8* was significantly higher in ‘Golden Delicious’ than in ‘Jinhong,’ and with the continuous decrease in temperature, its expression level showed a significant downregulation. *MdPRMT9*, *MdPRMT10*, *MdPRMT11*, and *MdJMJ23* had higher average expression levels in ‘Golden Delicious’ than in ‘Jinhong’, but their expression levels did not show an obvious pattern of change. It is worth noting that *MdJMJ19* had very high expression levels at 4°C in both apple cultivars, and as the temperature decreased, its expression levels were significantly downregulated. In particular, *MdJMJ19* in ‘Golden Delicious’ showed a stable expression level at -9°C and even lower temperatures, while in ‘Jinhong’, a slight upregulation was observed starting from -4°C, and although the expression levels decreased at -14°C and -29°C, the overall trend was still upregulation. In ‘Jinhong’, the expression level of *MdJMJ15* was significantly higher at -4°C and lower temperatures than at 4°C, while in ‘Golden Delicious’, the change in expression levels was not significant. *MdPRMT2* and *MdPRMT13* had significantly higher expression levels at -4°C in ‘Jinhong’ than at 4°C, and their expression levels were downregulated at lower temperatures, which was not observed in ‘Golden Delicious’. *MdJMJ18* showed an upregulation of expression levels between -4°C and -14°C in both cultivars, and its expression levels decreased at even lower temperatures, indicating that this gene may play a role in apple’s resistance to low temperature.

Drought is also one of the main types of stress faced during apple cultivation, especially in drought-prone areas of China (Berger et al., 2016). We analyzed the RNA-seq data of ‘GL3’ under drought treatment (SRP347250) (**Figure 7B**). The analysis results showed that compared to the control group, most *MdPRMTs* and *MdJMJs* showed varying degrees of upregulation under drought stress, with *MdPRMT2*, *MdPRMT6*, *MdPRMT14*, *MdJMJ18*, *MdJMJ13*, *MdJMJ20*, *MdJMJ21*, *MdJMJ25* and *MdJMJ27* exhibiting significant upregulation. Therefore, it can be inferred that these genes may be closely related to apple’s resistance to drought.

**Figure 8:**
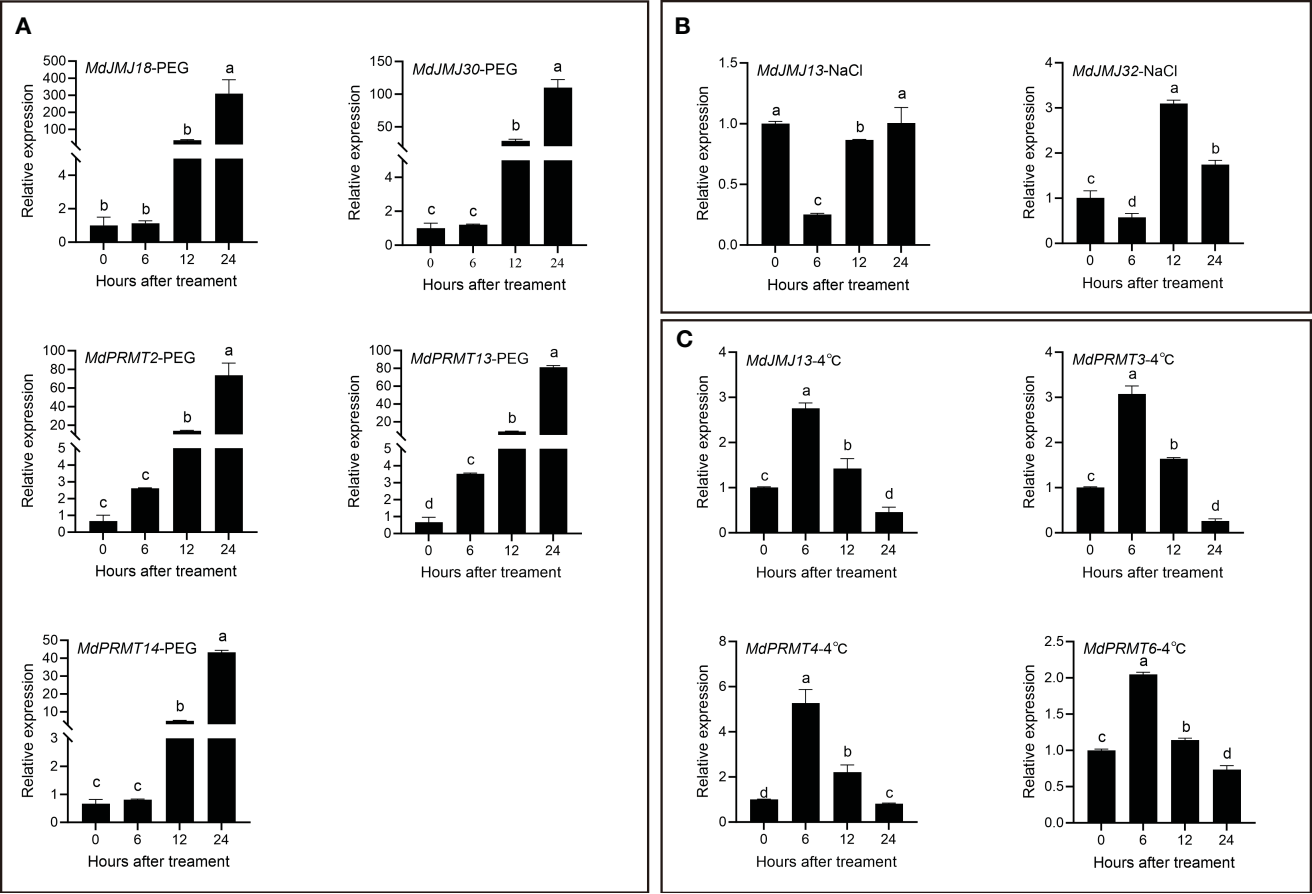
qRT-PCR analysis of selected *MdJMJ* and *MdPRMT* genes expression under PEG **(A)**, NaCl **(B)** and 4°C **(C)** treatment. 25 d-old leaves were treated with PEG, NaCl and 4°C for 0 h, 6 h, 12 h and 24 h The mean values ± SEM are shown for three biological replicates. Different letters above the bars indicate significant differences according to Duncan-test (*P* < 0.05).

We selected five *MdJMJ* and *MdPRMT* genes that were significantly upregulated under drought stress for qRT-PCR analysis. The results showed a significant and varying upregulation in all five genes upon PEG (as drought) treatment (**Figure 8A**). When two *MdJMJ* genes (*MdJMJ13* and *MdJMJ32*) were subjected to salt stress, both exhibited a decrease in expression after 6 h, followed by an increase at 12 h (**Figure 8B**). Similarly, the relative expression levels of the four genes under 4°C cold stress displayed a similar pattern, with an initial increase at 6 h and subsequent decline (**Figure 8C**).

### 3.7 Expression profiles of the apple *MdPRMTs* and *MdJMJs* in response to hormone treatment

**Figure 9:**
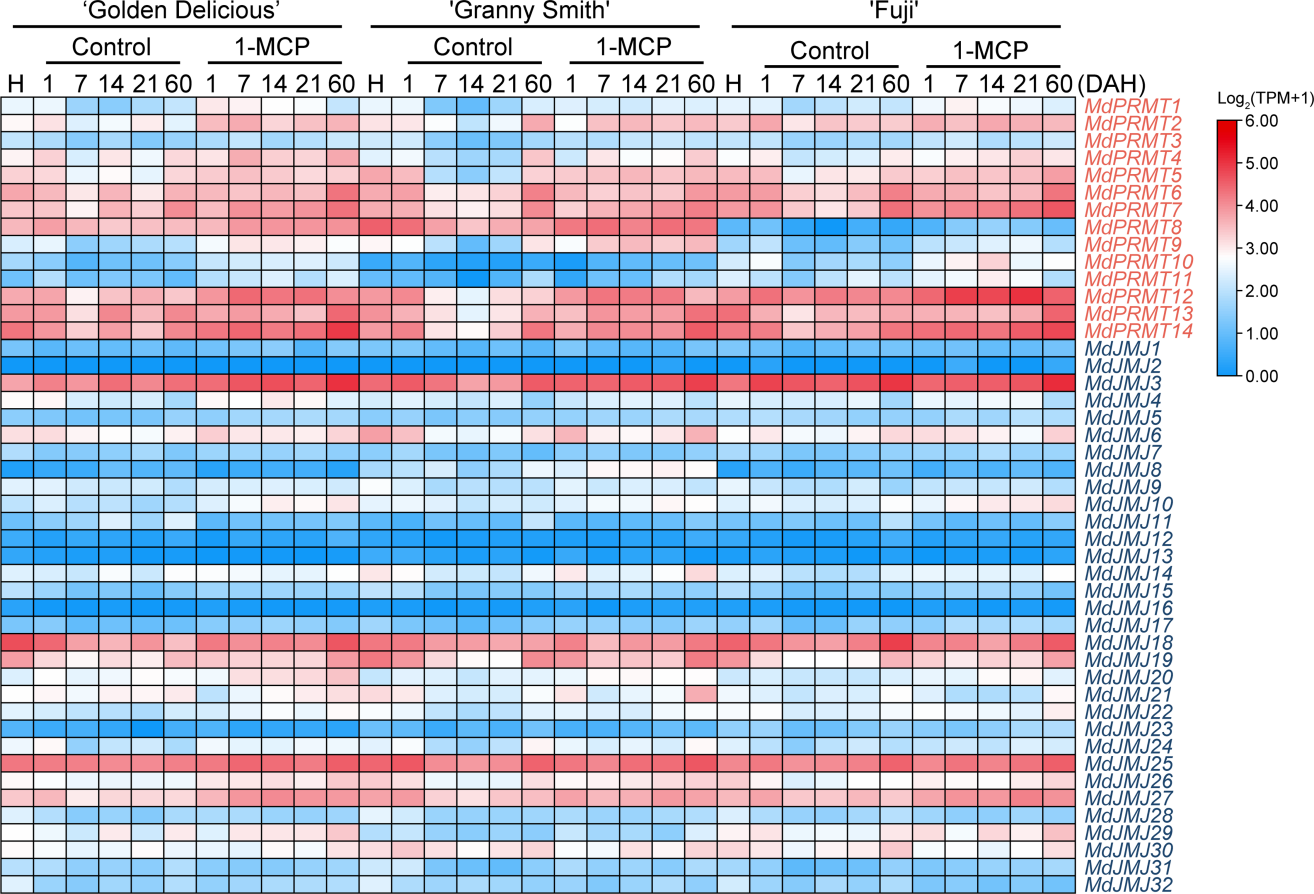
Expression profiles of the apple *MdPRMT* and *MdJMJ* genes during fruit response to 1-MCP treatment (SRP334206). Different shades of red and blue denote the extent of the expression values according to the color bar provided [log_2_(TPM+1)].

Plant hormones play a crucial role in the growth and development of plants. 1-MCP (1-Methylcyclopropene) is commonly used for preserving ethylene-producing or ethylene-sensitive fruits, effectively delaying fruit senescence (Berger et al., 2016). We analyzed the RNA-seq data (SRP334206) of three apple fruit varieties (‘Golden Delicious’, ‘Granny Smith’, and ‘Fuji’) treated with 1-MCP (**Figure 9**). The analysis revealed that *MdPRMT2*, *MdPRMT7*, *MdPRMT12*, *MdPRMT13*, and *MdPRMT14* showed significantly upregulated expression in the 1-MCP treatment group compared to the control group in all three apple varieties. Additionally, the expression of *MdPRMT8* in ‘Fuji’ was significantly lower than the other two varieties. Similarly, the expression of *MdJMJ3*, *MdJMJ18*, *MdJMJ25*, and *MdJMJ27* was significantly higher in the 1-MCP treatment group, with *MdJMJ3* showing the most noticeable upregulation at 60 d after 1-MCP treatment. This suggests that the aforementioned genes may be important for regulating the response of apple fruits to 1-MCP.

**Figure 10:**
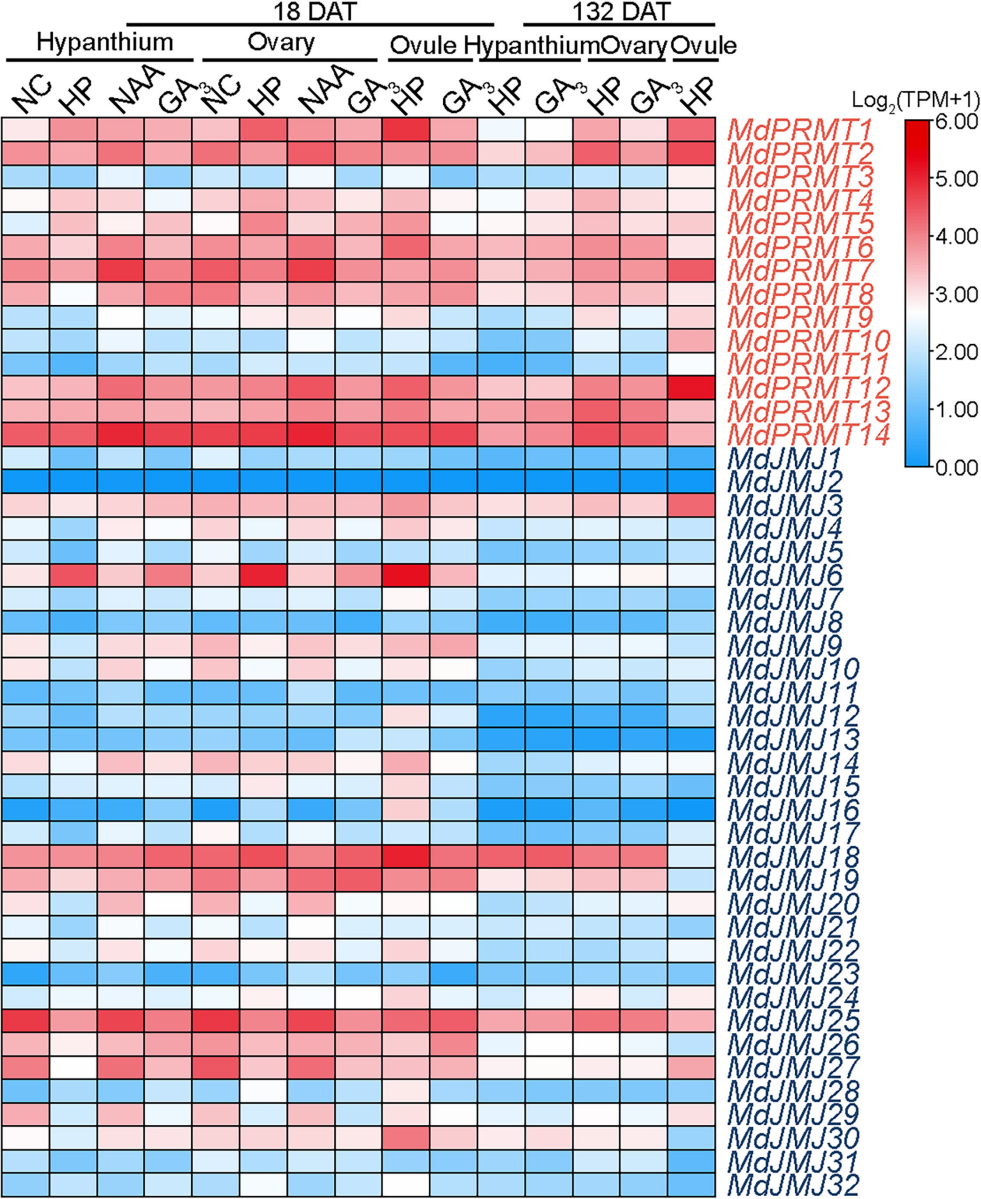
Expression profiles of the *MdPRMT* and *MdJMJ* genes in response to flower treatments with NAA and GA_3_ in apple (SRP185711). Different shades of red and blue denote the extent of the expression values according to the color bar provided [log_2_(TPM+1)].

GA_3_ and NAA are important plant hormones involved in various plant growth and development processes (Hennerty and Forshey, 1972; Pharis and King, 1985). We analyzed the RNA-seq data (SRP185711) of the main floral organs of ‘Honeycrisp’ apples after 18 and 132 days of NAA and GA_3_ treatment. The analysis showed that the expression of *MdJMJ6* in the hypanthium, ovary, and ovule was significantly lower after 18 days of NAA and GA_3_ treatment compared to the hand-pollinated control (HP). Additionally, after 132 days of GA_3_ treatment, all three organs showed lower expression levels of *MdJMJ6*. *MdPRMT8* and *MdJMJ19* exhibited significant upregulation in expression after 18 days of NAA and GA_3_ treatment compared to HP. Furthermore, the expression levels of *MdPRMTs* and *MdJMJs* in all organs were significantly lower after 132 days of GA_3_ treatment compared to 18 days (**Figure 10**).

Finally, we also investigated the response of apple plants at different growth and development stages to Indole-3-butyric acid (IBA), which promotes adventitious root formation (**Supplementary Figure S1**). The ability of apple plants to generate adventitious roots varies at different stages of growth and development. We analyzed the RNA-seq data (SRP330812) of adult and juvenile apple plants treated with IBA. Most of the *MdPRMTs* and *MdJMJs* showed no significant sensitivity to IBA treatment. However, it should be noted that in adult plants, the expression of *MdJMJ19* was upregulated after 6 and 12 hours of IBA treatment compared to mock, followed by a decrease with longer treatment time. In juvenile plants, except for the 12-hour treatment, the expression of *MdJMJ19* was higher than mock.

## 4 Discussion

**Figure 11:**
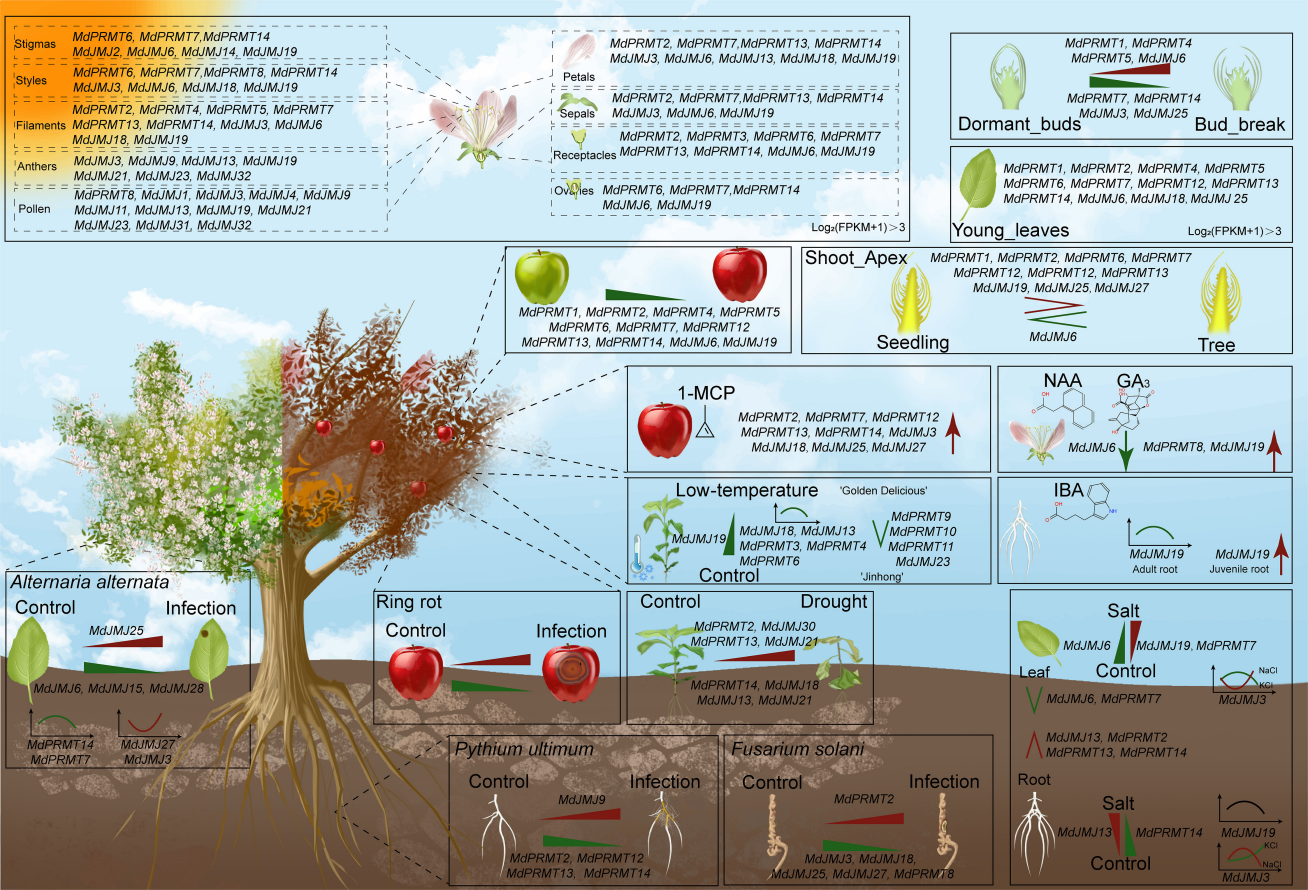
The role of the *MdJMJ* and *MdPRMT* genes in apple.

Epigenetics involves stable heritable variations in organisms without changes in DNA sequences, including histone modifications (Berger et al., 2009). Histone modifications play roles in maintaining genome stability, gene regulation, and cellular processes. Research on histone modifications in important crops like rice (Ahmad et al., 2011) and tomato (Li et al., 2020) has shown their impact on gene expression and cellular activities. In this study, *MdJMJ* and *MdPRMT* families in apples were identified, characterized, and analyzed for potential roles in stress response, hormone regulation, and tissue development (**Figure 11**).

In order to better understand the phylogenetic relationship between the *JMJ* and *PRMT* gene families in apple and *Arabidopsis*, we constructed a phylogenetic tree. Based on the results of related studies in *Arabidopsis*, we classified the *JMJ* gene family into corresponding subfamilies, and found that the *JMJ* genes clustered in a logical manner. Similarly, the *PRMT* genes also showed reasonable clustering. Gene duplication plays a crucial role in species evolution (Panchy et al., 2016). In the current apple reference genome, we observed that most of the *MdJMJ* and *MdPRMT* genes have undergone gene duplication. In this study, we used the *Arabidopsis* genome as an outgroup and employed Dupgen_finder to identify the types of gene duplication within the *MdJMJ* and *MdPRMT* gene families. The results indicated that apple has recently experienced a whole-genome duplication event (Qiao et al., 2019), the predominant types of gene duplications in the *MdJMJ* and *MdPRMT* families following whole-genome duplication were WGD, with a smaller proportion of PD, TRD and TD types. Generally, different categories of gene duplication exhibit distinct patterns of functional evolution. The Ka/Ks values for most homologous gene pairs in the *MdJMJ* and *MdPRMT* gene families were less than 1, suggesting they have undergone purifying selection, eliminating deleterious mutations. Notably, a cluster of *MdPRMT* genes was identified on apple chromosome 15. Analysis showed that these genes were mainly involved in proximal repeats. In the promoter regions, significant differences in the types of *cis*-regulatory elements were observed between the PD-pair (*MdPRMT9*-*MdPRMT11*, *MdPRMT9*-*MdPRMT10*). In terms of expression profiles, *MdPRMT9* exhibited higher expression levels in most parts of apple flowers compared to *MdPRMT10* and *MdPRMT11*, under various treatments including biotic stress, cold stress and 1-MCP treatment. This suggests that TD-pairs retain higher conservation in their structural homogeneity, without affecting their functional differentiation (Qiao et al., 2019), while the WGD duplication serves as the main driver for the expansion of the *MdJMJ* and *MdPRMT* gene families, contributing to the diversity in family member structures (Feng et al., 2024).

We particularly focused on the differences in *cis*-regulatory elements between TD and PD repeats in the *MdJM*J and *MdPRMT* gene families. For example, between the TD-pair (*MdJMJ1*-*MdJMJ2*), 14 types of cis-acting elements were identified, but only 7 of these were shared by both genes. A similar situation was also observed between the two PD-pairs in the *MdPRMT* family. This suggests that tandem duplication and proximal duplication play a significant role in the evolution of novel functions in the *MdJMJ* and *MdPRMT* gene families (Cannon et al., 2004). Furthermore, a complementary pattern of *cis*-regulatory elements is observed in the promoter regions of WGD gene pairs. For example, *MdJMJ20* and *MdJMJ26* form a WGD duplicate pair with *MdJMJ9*, and among the predicted 14 *cis*-elements, only four (Light, MYB_drought, ABRE, and ARE) are shared by all three genes. The above results can be explained by the duplication-degeneration-complementation (DDC) model. The DDC model suggests that following a gene duplication event, two alleles at two different loci experience degeneration mutations at different times, leading to a pair of duplicate genes sharing the ancestral gene’s functions and completing the ancestral gene’s functions in a complementary manner (Hahn, 2009). Comparative analysis of gene expression in various apple tissues revealed significant tissue-specific expression differences among most duplicate gene pairs (**Figure 6**), suggesting that these genes have evolved by differentiating ancestral gene functions or expression patterns under different tissue conditions. Additionally, we focused on comparing *cis*-regulatory elements in the promoters of apples (*MdJMJ* and *MdPRMT* families) with those of *Arabidopsis* (*AtJMJ* and *AtPRMT* families) (**Supplementary Figure S2**). The average number of *cis*-elements in apples is 9.28 for *MdJMJ* and 9.57 for *MdPRMT*, compared to 7.76 for *AtJMJ* and 8.22 for *AtPRMT* in *Arabidopsis*. The differences in element types can be attributed to both the high species specificity of *cis*-elements and the result of different selection pressures and environmental conditions acting on the ancestral genes after duplication events, leading to the acquisition of new *cis*-regulatory elements in their promoters (Zhang, 2003; Lan and Pritchard, 2016). The genes in model organisms generally have more comprehensive functional studies. For example, the homologous gene of *MdJMJ21*, *AtJMJ25*, has been confirmed to be involved in the formation of the very early embryo and endosperm in *Arabidopsis* (Day et al., 2008). We found that there is an endosperm-related *cis*-acting element in the promoter region of both *MdJMJ21* and *AtJMJ25* (**Supplementary Figure S2**), suggesting that *MdJMJ21* may be involved in the regulation of apple endosperm formation. The *Arabidopsis* gene *AtLDL1* has been confirmed to participate in physiological (defense) and developmental (flowering time) processes (Dutta et al., 2017), possessing ARE, ABRE, and Stress_defense-related regulatory elements. Homologous to *AtLDL1*, *MdJMJ3* has eight ABRE and one ARE regulatory elements and shows up-regulated expression under drought stress treatment, and it is significantly expressed in anthers and pollen. Therefore, *MdJMJ3* may similarly participate in the regulation of apple abiotic stress and flower development processes. Further homologous analysis of some genes in the *MdJMJ* and *MdPRMT* gene families with model organisms can reveal their potential functions.

Multiple models have been proposed to elucidate the retention and evolution of gene duplications, including gene dosage balance (Birchler et al., 2005), subfunctionalization (SF), neofunctionalization (NF) (Sandve et al., 2018), expression specialization, and pseudogenization (Qiao et al., 2019). Based on expression profiles, we studied the expression differences between duplicated genes and explored the mechanisms of gene retention. Interestingly, at different stages of fruit development, many gene pairs showed complementary expression patterns, which may indicate subfunctionalization. For instance, the gene pair *MdPRMT1* and *MdPRMT12*, where one gene copy had higher expression levels at several stages while the other gene copy had higher expression levels at the remaining stages. Some gene pairs exhibited parallel expression patterns, suggesting that gene dosage balance is imposed in the evolution of duplicated gene pairs to maintain the total expression level of ancestral genes. For example, duplicated gene pairs like *MdPRMT9*-*MdPRMT10* and *MdJMJ1*-*MdJMJ2* showed similar expression patterns at different stages of fruit development. Additionally, some gene pairs showed expression specialization and nonfunctionalization, such as *MdJMJ19*-*MdJMJ31* and *MdJMJ18*-*MdJMJ30*, where one gene copy was highly expressed at almost all stages of dormant buds, while the other gene copy showed low or no expression. We primarily focused on the performance of *MdJMJ* and *MdPRMT* under abiotic stress. It was found that some duplication gene pairs exhibited similar expression patterns under abiotic stress treatments, such as *MdJMJ18*-*MdJMJ30* and *MdJMJ13*-*MdJMJ32*, where qPT-PCR and transcriptome analyses confirmed their consistent expression patterns under drought stress and salt stress, and a certain number of related *cis*-regulatory elements were identified in their promoter regions, possibly involved in regulating gene expression under stress conditions. These results demonstrate the complexity of the expansion and evolution of the *MdJMJ* and *MdPRMT* gene families, laying the foundation for further elucidating their more specific regulatory mechanisms.

## 5 Conclusions

In this study, a total of 14 *MdPRMT* genes and 32 *MdJMJ* genes were identified in apples. Co-linearity analysis showed that both gene families exhibited high conservation between *Arabidopsis* and apple. Promoter analysis indicated that *MdPRMTs* and *MdJMJs* may play important roles in plant growth and development, light response, hormone response, and stress response. Based on the analysis of transcription levels of *MdPRMTs* and *MdJMJs* in different tissues and developmental stages, we found that *MdPRMTs* and *MdJMJs* may have multiple functions in the growth and development process of apples. Especially, *MdPRMT13*, *MdPRMT14*, and *MdJMJ19* showed a downtrend during fruit ripening and aging process. *MdPRMT7*, *MdPRMT12*, *MdJMJ3*, *MdJMJ13*, *MdJMJ21*, *MdJMJ23*, *MdJMJ25*, *MdJMJ26*, *MdJMJ27*, and *MdJMJ32* exhibited high expression levels in specific organs and tissues of apples. *MdJMJ25*, *MdPRMT7*, *MdPRMT14*, *MdJMJ9*, *MdPRMT2*, *MdJMJ3*, *MdJMJ18*, *MdJMJ27*, and *MdPRMT8* were involved in response to biotic stress. *MdPRMT2*, *MdPRMT7*, *MdPRMT12*, *MdPRMT13*, and *MdPRMT14* showed a significant upregulation in the 1-MCP-treated group of three apple varieties. *MdPRMT8* and *MdJMJ19* exhibited significant upregulation within 18 days after NAA and GA_3_ treatment. *MdJMJ19* may be associated with apple response to IBA.

## Data availability statement

The datasets presented in this study can be found in online repositories. The names of the repository/repositories and accession number(s) can be found below: https://www.ncbi.nlm.nih.gov/, SRP034165, SRP050139, SRP0999578, SRP125281, SRP334206, SRP185711, SRP229388, SRP347250, SRP091754, SRP048684, SRP239526, SRP153065, SRP330812, https://ngdc.cncb.ac.cn/, CRA002596.

## Author contributions

SS: Conceptualization, Data curation, Formal analysis, Investigation, Methodology, Software, Validation, Visualization, Writing – original draft, Writing – review & editing. MJ: Data curation, Formal analysis, Investigation, Software, Validation, Writing – review & editing. JC: Formal analysis, Investigation, Validation, Writing – review & editing. MZ: Investigation, Software, Validation, Writing – review & editing. XX: Conceptualization, Funding acquisition, Resources, Writing – review & editing. CC: Conceptualization, Formal analysis, Project administration, Resources, Software, Supervision, Writing – review & editing.

## Supplementary material

Supplementary Table 4 Orthologous gene pairs between apple, Arabidopsis, rice and zea mays JMJ and PRMT Genes.
