# Supplementary material for: Genome-wide identification and expression analysis of protein arginine methyltransferase and JmjC domain-containing family in apple
Source: Front Plant Sci. 2024 May 28;15:1381753. doi: 10.3389/fpls.2024.1381753 (PMC11165092; doi:10.3389/fpls.2024.1381753)
Supplement: Supplementary file 2 [file DataSheet_2.docx]

**Supplementary Information**

Title: Genome-Wide Identification and Expression Analysis of *Protein Arginine Methyltransferase* (*PRMT*) and *JmjC Domain-Containing* Family (*JMJ*) in Apple

**Authors:** Shenghui Su, Min Ji, Jiaqi Chen, Meidie Zhang, Yongbing Yuan, Jiyun Nie, Xiaozhao Xu*, Chenxia Cheng *

**Institution for all authors:** College of Horticulture, Qingdao Agricultural University, 700 Changcheng Road, Qingdao 266109, Shandong, China.

* Correspondence: [201801006@qau.edu.cn](mailto:201801006@qau.edu.cn); [chengchenxia@qau.edu.cn](mailto:chengchenxia@qau.edu.cn);

**Submitting author:** Xiaozhao Xu.


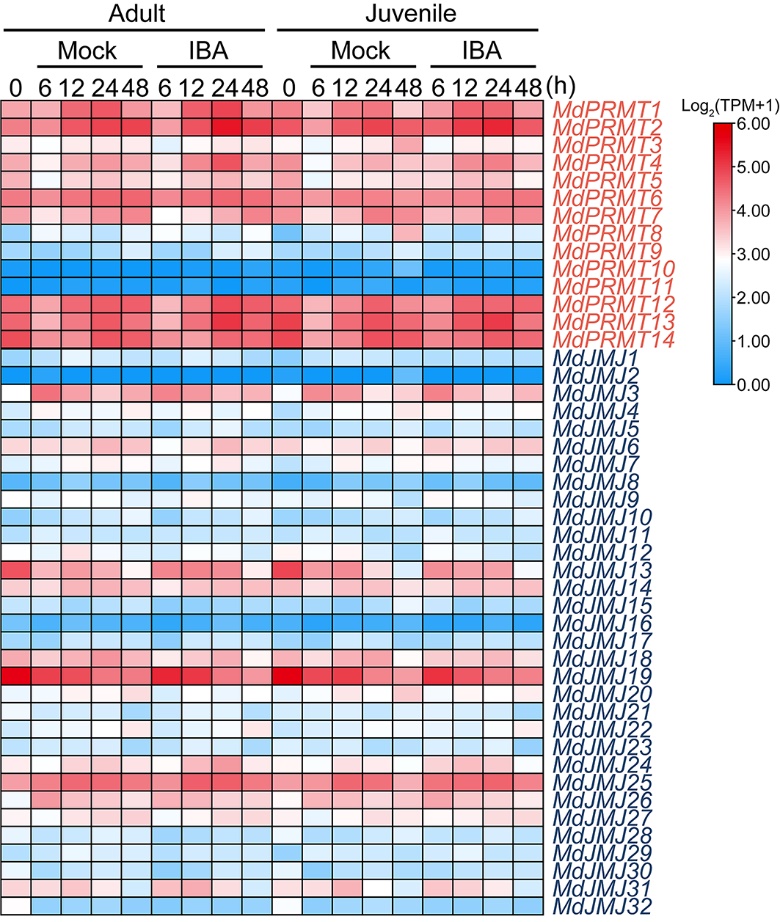


**Figure S1.** Expression profiles of the apple *MdPRMT* and *MdJMJ* genes during root response to IBA treatment (SRP330812). Different shades of red and blue denote the extent of the expression values according to the colour bar provided (log_2_(FPKM+1)).


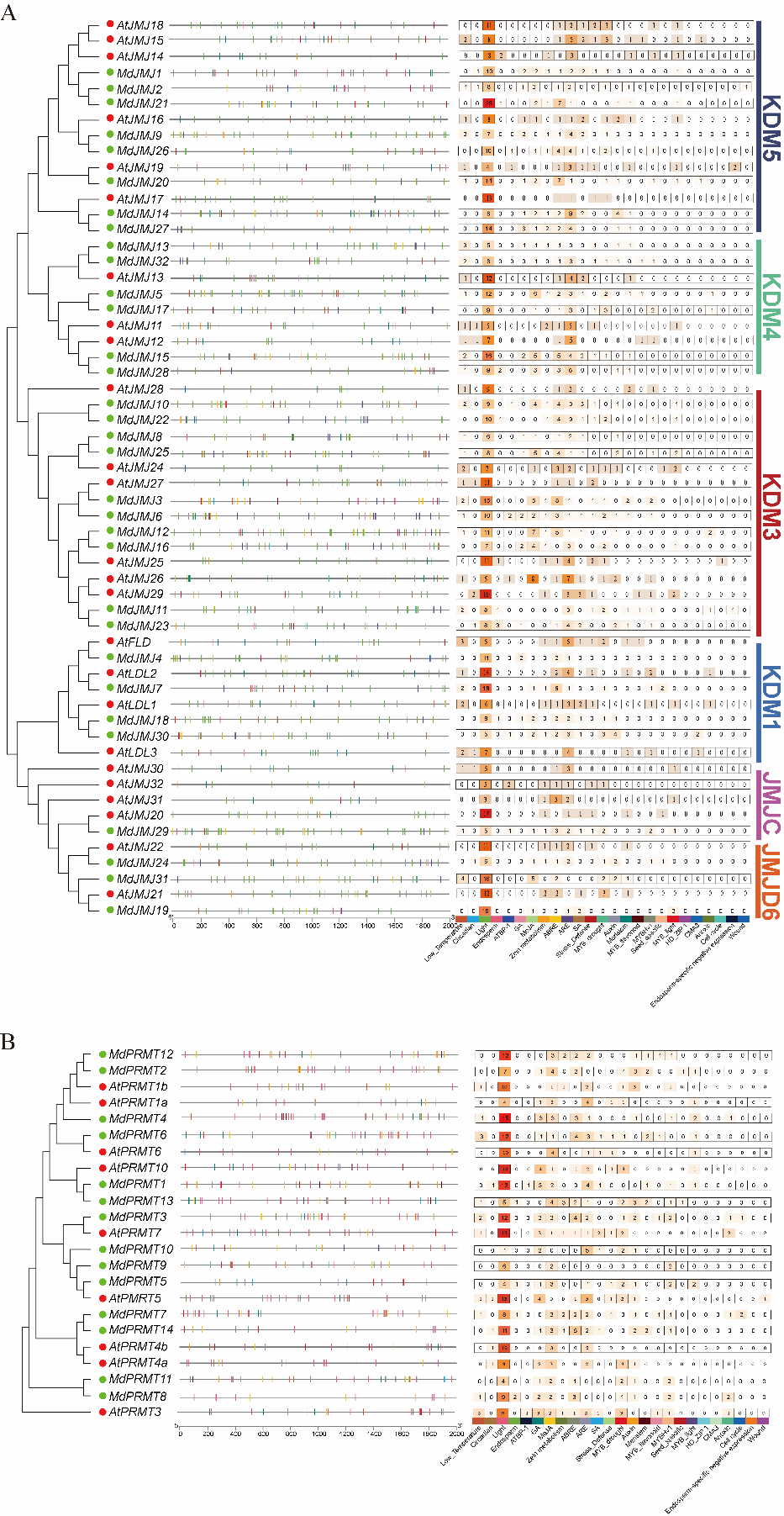


Figure S2. The *cis*-elements analysis of *JMJ* and *PRMT* promoters in apple and *Arabidopsis*. (**A**) The distribution of *cis*-elements in the promoter of each *JMJ* genes. (**B**) The distribution of *cis*-elements in the promoter of each *PRMT* genes.
